# Pilot testing of a brief pre-consultation medicines adherence tool in a geriatric outpatient setting

**DOI:** 10.1093/ageing/afag128

**Published:** 2026-05-17

**Authors:** Jennifer M Stevenson, Massimo Pezzolato, Sarah Chapman, Aiman Ibrahim, Tae Ibrahim, Nabeel Syed, John A Weinman

**Affiliations:** King's College London – Pharmacy Department, Institute of Pharmaceutical Science, London, United Kingdom of Great Britain and Northern Ireland; Guy's and St Thomas' NHS Foundation Trust - Pharmacy Department, London, England, United Kingdom of Great Britain and Northern Ireland; Applied Research Division for Cognitive and Psychological Science, European Institute of Oncology, Milan, Italy; University of Milan - Department of Oncology and Hemato-oncology, Milan, Italy; King's College London – Pharmacy Department, Institute of Pharmaceutical Science, London, United Kingdom of Great Britain and Northern Ireland; King's College London - GKT School of Medical Education, London, United Kingdom of Great Britain and Northern Ireland; King's College London - GKT School of Medical Education, London, United Kingdom of Great Britain and Northern Ireland; Great Ormond Street Hospital for Children NHS Foundation Trust - Department of Pharmacy, London, United Kingdom of Great Britain and Northern Ireland; King's College London – Pharmacy Department, Institute of Pharmaceutical Science, London, United Kingdom of Great Britain and Northern Ireland

**Keywords:** medication adherence, older adults, outpatients

## Abstract

**Introduction:**

In older adults, medication non-adherence is prevalent and harmful. Current methods of identification have limitations, with direct questioning often being met with a reluctance to ‘admit’ non-adherence to healthcare professionals. The *Making Medicines Work for You (MMWFY)* tool consists of seven items (mapped to recognised adherence barriers) and a free-text box, and has been developed to support patients and clinicians identify and discuss adherence issues in a clinical setting. This study piloted the tool in a geriatric outpatient population.

**Methods:**

Patients attending the Older Person’ Assessment Unit at a London teaching hospital completed the MMWFY tool. Descriptive analysis was used to determine the incidence and type of potential adherence barriers. Associations and correlations between the tool and existing measures of adherence (MARS-5) and medicines beliefs (BMQ) were assessed.

**Results:**

Of 245 patients approached, 226 consented to participate: 120 (53.1%) males; mean age 78 years. On the MMWFY, 115 (50.9%) participants selected at least one item (median 1, range 0–5), indicating the presence of a potential adherence barrier. ‘*I’ve found my own way to use my medicines*’ (*n* = 53, 27.5%) and *‘I sometimes forget to take my medicines*’ (*n* = 45, 23.3%) were the most frequently selected. The tool demonstrated association and correlation with validated adherence measures.

**Conclusion:**

Half of the participants reported potential barriers to medication adherence. The high rate of completion and association with existing adherence measures suggest that the tool may have value in this setting. Further work is required to explore the barriers identified and how to use these to develop appropriate adherence support.

## Key Points

- Medication non-adherence is highly prevalent in older adults but often goes undetected.
- The range of factors influencing medication non-adherence is not routinely considered.
- A brief pre-consultation questionnaire could support the personalisation of adherence interventions

## Introduction

In older adults, the prevalence of medication non-adherence is 50%, and is attributed to 10% of hospital admissions [1], and almost a quarter of post-discharge medication-related harm [2]. Cognitive and sensory impairments, and polypharmacy, increase the risk of non-adherence [3–5]. Frailty, which reduces an individual’s ability to adjust to stressors like medication changes, heightens susceptibility to the harmful effects of medication non-adherence [6].

Assessment of medication adherence is part of the Comprehensive Geriatrics Assessment (CGA) [7, 8], yet existing measures [9] are not routinely used in clinical practice or CGA guidance. Existing measures do not support the identification of barriers to adherence, and patients report a reluctance to admit to non-adherence when asked directly [10], risking potential underreporting. The *Making Medicines Work For You* (MMWFY) tool was developed to identify issues that may impact adherence. Clinicians can then explore these issues to inform adherence support. Current interventions to overcome medication issues are frequently not personalised, often focusing on a limited range of issues. Interventions are used without considering all the factors influencing non-adherence [11–13]. Initial studies of the MMWFY tool demonstrate good usability and validity [14], and the tool has been embedded in electronic health records, illustrating potential scalability. However, it has not been evaluated in an older population.

This study piloted the MMWFY tool in a geriatric outpatient population to: (i) Determine the prevalence and types of adherence barriers to provide insight into the range of potential issues, which will inform the type of interventions required; (ii) explore the acceptability and usability of the MMWFY tool, and (iii) determine the validity of the tool through evaluation of the relationship between adherence and beliefs measures and the tool, and between adherence barriers and cognitive impairment, frailty, and support needs.

## Methods

Patients aged 65 years and over attending an Older Persons’ outpatient department (OPAU) (June–October 2022) completed a paper form of the MMWFY tool, with carer or researcher support where necessary. Patients were greeted using a scripted approach and provided with written information outlining the aims of the MMWFY tool.

### M‌MWFY tool

Developed by the Centre for Adherence Research and Education (CARE) at King’s College London, the MMWFY tool facilitates assessment of adherence barriers through a tick box questionnaire. [14] Co-designed with patients and clinicians, it allows patients to identify factors influencing their adherence without the discomfort of directly admitting non-adherence. An 8-item questionnaire, items 1–7 map broadly onto barriers classified by the COM-B framework for adherence [15]. Each item selected is scored 1, giving a score range from 0 to 7. Item 8 is a free-text box that allows patients to report any other issues.

### Other assessments

Patients also completed: two established adherence measures, the 5-item *Medication Adherence Report Scale* (MARS-5) (>23 = adherent) [16] and a modified *Beliefs about Medicines Questionnaire* (BMQ) assessing Necessity and Concern beliefs [17, 18]. Patient demographics, including age, gender, clinic attended and frailty scores (Clinical Frailty Score (CFS) [19], Edmonton Frailty Score (EFS) [20]), cognitive impairment (Montreal Cognitive Assessment (MoCA) [21], Clock Drawing Test (CDT) [22], a 6-item Cognitive Impairment Test (CIT) [23], Mini-Mental State Examination (MMSE) [24] and Addenbrooke’s Cognitive Examination III (ACE-III) [25]), use of a dosette box, and independence with medicines management were collected. To understand the usability of the MMWFY tool, the need for assistance to complete MMWFY tool and suggested improvements were captured using yes/no responses and free-text boxes. Patients were excluded if they were <65 years or consent was not gained. Where consent was gained via a relative/carer, e.g. due to cognitive impairment, or the participant had limited English, the relative/carer supported completion of the tool.

### Statistical analyses

Frailty and cognitive impairment were dichotomised: frailty: CFS ≥5; EFS ≥8; and cognitive impairment: CDT ≤ 2 [22]; MOCA<26 [21]; 6CIT ≥ 8 [26]; MMSE≤24 [27]; ACE-III ≤ 88 [25]. Spearman’s rank correlation, Chi-squared test with Yates’ continuity correction, Fisher’s exact test and Mann–Whitney *U* tests were used to assess associations between variables.

### Approvals

As part of a medicines optimisation improvement programme, using an existing tool in use within the hospital site, this project was considered a service evaluation. Research Ethics approval was not required, as confirmed by the NHS Health Research Authority Decision Tool (Is my study research?)—the standard tool used in the UK (Supplementary File 1). Local Research and Development approval was obtained (Project ID: 13719).

## Results

A total of 245 patients were approached; 226 (92%) consented to participate. Participants’ characteristics are outlined in Table 1.

**Table 1:** Characteristics of participants.

Characteristic (*n* = 226)	Frequency (%)
Age, median (range) (years)	78 (66–99)
Sex (female)	106 (46.9)
Frailty^a^	26 (36.1)
Cognitive impairment^b^	46 (49.5)
Support with medicines	64 (28.3)
Dosette box	67 (29.6)
Clinic	
*CGA (HF, PD, general)*	46 (20.4)
*GOLD*	52 (23.0)
*Others*	69 (30.5)
*POPS*	59 (26.1)

Clinic (Others): Infusion, Bowel/Bladder, CNS, Dietician, Memory, Physio, TWOC​, Falls, Bone Health; CGA: Comprehensive Geriatric Assessment; GOLD: Geriatric Oncology Liaison; HF: Heart Failure; PD: Parkinson’s Disease; POPS: Perioperative Older Persons Service​

^a^Frailty measures and frail cut points: CFS ≥5; EFS ≥8 (data available for *n* = 72, 31.9% participants)

^b^Cognitive function measures and impaired cut points: CDT ≤2; MoCA<26; 6CIT ≥ 8; MMSE≤24; ACE-III ≤ 88 (*n* = 93)​

### Prevalence and types of personal adherence barriers

**Figure 1:**
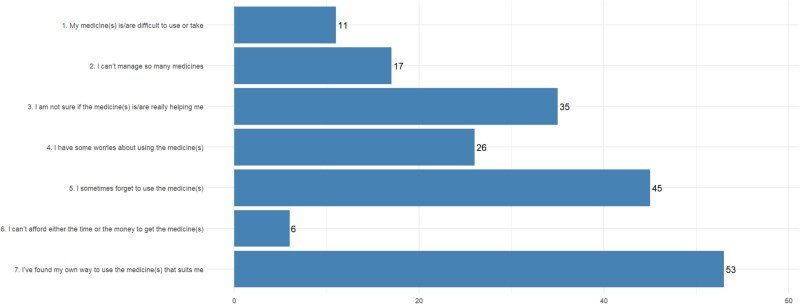
MMWFY item frequency.

Over half of the participants (*n* = 115, 50.9%) selected at least one adherence barrier (range 0–5). Every barrier was selected by at least one participant (Figure 1): *‘I’ve found my own way to use my medicines*’ (*n* = 53, 23.4% of participants, 27.5% of selected items) and ‘*I sometimes forget to take my medicines’* (*n* = 45, 19.9% of participants, 23.3% of selected items) were the most frequently selected.

### M‌MWFY acceptability and usability

The majority of participants were happy to complete the tool (92%), with assistance required by 137 (60.6%) (e.g. questions were read aloud as the participant forgot their glasses), while 37 (16.4%) thought the tool could be improved (e.g. the wording of item 7 was confusing).

Participants who required assistance to complete the tool selected more barriers compared to those who did not require assistance, (*U* = 5166.5, *P* = .036). Participants needing assistance were more likely to select “4. *I have some worries about using the medicine(s)*” (*P* = .032; OR = 1.112; CI 95% [0.097; 2.127]), “5*. I sometimes forget to use the medicine(s*)” (*P* = .026; OR = 0.850; CI 95% [0.110; 1.591]), and “8. *Do you have any questions or things you’d like to discuss about your medicines today?*” (*P* = .015; OR = -0.921; CI 95% [−1.647; −0.195]).

Participants were more likely to need assistance if they were living with cognitive impairment (χ^2^ = 13.212, *P* < .001) or frailty (χ^2^ = 10.174, *P* = .001).

### M‌MWFY validity

#### Relationship to adherence and beliefs measures

Patients who indicated more barriers had lower adherence as assessed by MARS-5 (rho = −0.34, *P* < .001). A stronger association with MARS-5 was demonstrated when two or more barriers were selected (χ^2^ = 23.032, *P* < .001) compared to one or more (χ^2^ = 8.531, *P* = .003).

Significant correlations were observed for both BMQ dimensions: the greater the number of barriers selected, the lower perceived necessity of medicines (rho = −0.17, *P* = .010) and the greater the concerns about medicines (rho = 0.292, *P* < .001).

#### Relationship between MMWFY responses and cognitive impairment, frailty and support needs

Participants with cognitive impairment selected more items than participants without cognitive impairment (*U* = 733.5, *P* = .005). Participants with cognitive impairment were more likely to select ‘2. I can’t manage so many medicines’ (*P* = .015; OR = 2.270; CI 95% [0.148; 4.393]), and ‘3. I am not sure if the medicine(s) is/are really helping me’ (*P* = .022; OR = 1.528; CI 95% [0.177; 2.880]).

Participants using a dosette box were more likely to select at least one item (χ^2^ = 3.951, *P* = .047) and have higher concerns compared to those not using one (*U* = 4373, *P* = .044). No significant difference was observed in the BMQ ‘necessity’ dimension.

No significant results were found when the aforementioned analyses were applied to groups defined by the presence or absence of frailty.

## Discussion

More than half of patients attending the OPAU report potential barriers to medication adherence that are not adequately addressed by current interventions. The MMWFY tool demonstrated acceptability, usability and validity.

### Self-reported adherence barriers

*‘I’ve found my own way to use my medicines that suits me*’ was the most prevalent. While participants reported confusion around this item, which requires review with patients and carers, studies of patient’s lived experience with medications indicate that patients modify medication regimes in an attempt to manage the burden [28]: *‘If I’m going on a long trip on the bus, well I never take one (furosemide) in the morning because you have to keep going to the toilet…*.’ [29] Such coping strategies are important to consider if clinicians are to take a salutogenic approach to medicines management.

*‘I sometimes forget to take my medicines*’ was the second most frequently selected. This is perhaps unsurprising given that 49.5% of participants were living with cognitive impairment, a recognised factor influencing adherence [4]. Participants living with cognitive impairment were more likely to select at least one barrier compared to those without cognitive impairment. The most selected barriers by participants living with cognitive impairment were, ‘*I can’t manage so many medicines’* and ‘*I am not sure if my medicines are really helping me’*. These reflect the importance of considering barriers such as attention and beliefs, as well as ‘forgetfulness’ when supporting older adults with cognitive impairment to manage their medicines.

Across all participants, uncertainty relating to benefit (‘*I’m not sure if my medicines are really helping me’*) and concerns about medicines (‘*I have some worries about using my medicines’*) equated to 18% and 13% of barriers selected, respectively. Our findings support the theory that medicine adherence is influenced by a broad range of factors, not just physical and mental capability, but also patients’ beliefs and concerns about medicines [15].

### Future work

Building upon the feedback gathered, modifications to the tool should be made in collaboration with older adults and carers and should seek to improve independence in completion of the tool and translation into other languages. In its current format, 60% needed support, which may have implications for implementation in practice. Already embedded in EPIC®, completion of a digital version of the tool should be evaluated to determine its usability and potential for personalised intervention support.

## Conclusion

The MMWFY tool could support personalised discussions with older adults about their medications through the identification of influencing factors, in addition to cognitive and physical barriers, which are often the focus of standard adherence interventions. The high rate of completion and correlation with existing medication adherence and beliefs measures suggest that the MMWFY tool may have value in the geriatric outpatient setting. Further work is required to explore the barriers identified and to develop appropriate adherence support.

## Supplementary Material

aa-25-2833-File002_afag128
