# Supplementary figures and images for: Pilot testing of a brief pre-consultation medicines adherence tool in a geriatric outpatient setting
Source: Age Ageing. 2026 May 17;55(5):afag128. doi: 10.1093/ageing/afag128 (PMC13180272; doi:10.1093/ageing/afag128)

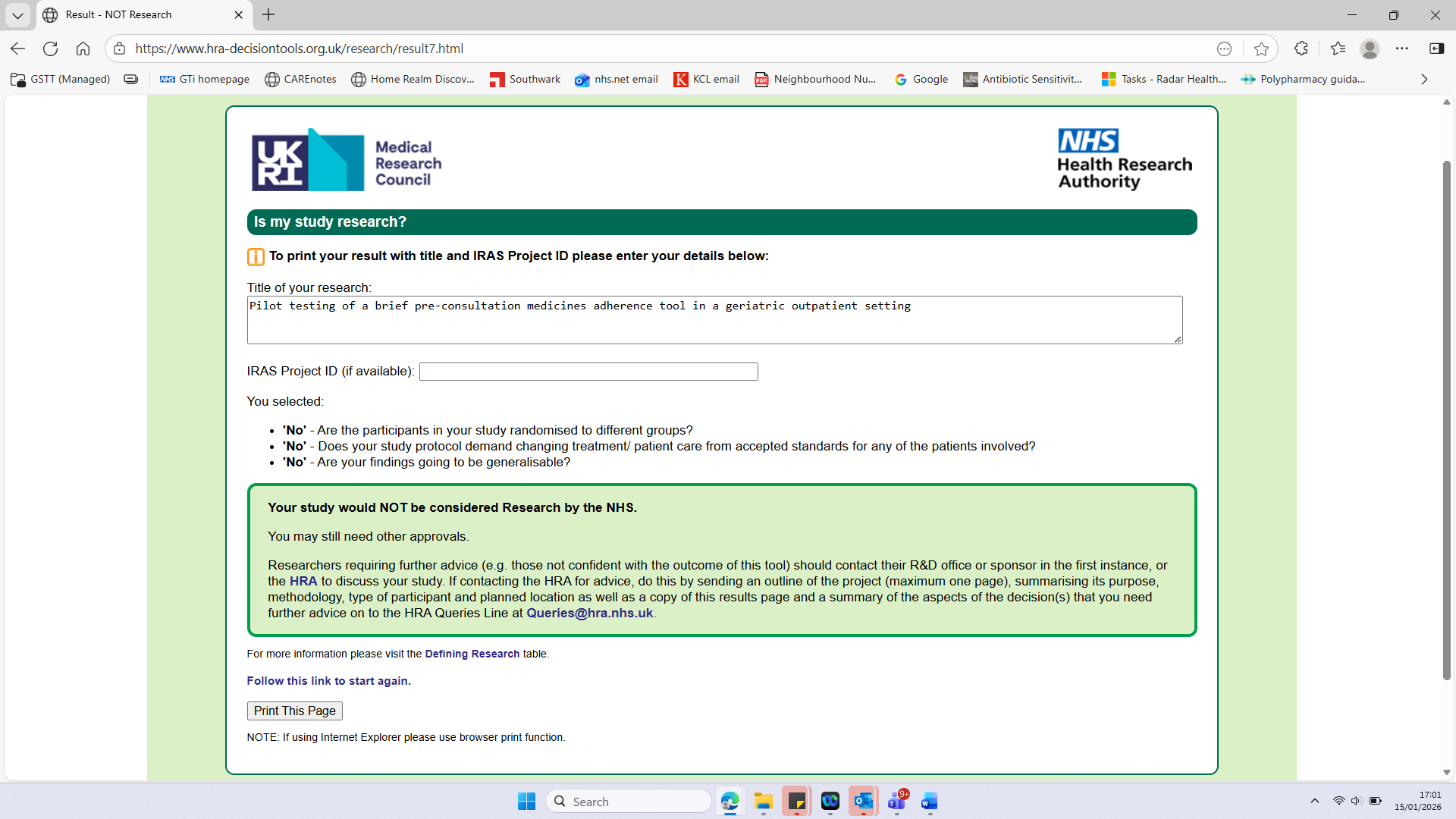

Supplement: aa-25-2833-File002_afag128 [file aa-25-2833-file002_afag128.docx]
